# Transactions—Ohio State Dental Society

**Published:** 1882-11

**Authors:** 


### Transactions.

The volume of transactions of the last annual meeting of the Ohio State Dental Society, containing one hundred and forty-nine pages, has just made its appearance. It contains the minutes, the papers read, and quite a full report of the discussions. The matter is all interesting, and much of it instructive. It was published under direction of the society by Dr. W. M. Herriott, of Indianapolis.

The work of the printer is well and neatly done, and the volume in all particulars is quite a respectable one.

Copies may be obtained of the editor of the REGISTER, or Dr. W. H. Sillito, of Xenia, O.

